# Commentary: Eosinophilic inflammation in hereditary angioedema: a single-center real-world retrospective chart review study

**DOI:** 10.3389/fimmu.2026.1836148

**Published:** 2026-05-07

**Authors:** Xiaomin Guo, Yangming Liu

**Affiliations:** 1 Department of General Medicine, Mianyang Central Hospital, School of Medicine, University of Electronic Science and Technology of China, Mianyang, China; 2 Department of General Medicine, Mianyang Sanjiang Hospital, Mianyang, China; 3 Department of Vascular Surgery, Mianyang Central Hospital, School of Medicine, University of Electronic Science and Technology of China, Mianyang, China

**Keywords:** biomarkers, bradykinin, eosinophil cationic protein, eosinophilic inflammation, hereditary angioedema

## 1 Introduction

Hereditary angioedema (HAE) has long been regarded as a rare disorder primarily driven by bradykinin-mediated increases in vascular permeability, clinically characterized by recurrent and unpredictable episodes of cutaneous or submucosal swelling that may become life-threatening when the upper airway is involved (1). Recently, Boch et al. (2), in a single-center real-world retrospective study, reported that patients with HAE exhibited elevated serum eosinophil cationic protein (ECP) levels despite no corresponding increase in absolute peripheral eosinophil counts, thereby suggesting a previously underappreciated eosinophilic inflammatory component in HAE. In that study, the authors included 48 patients with HAE and 1,880 controls, and applied Bayesian multilevel regression, inverse probability of treatment weighting, and post-stratification approaches to evaluate the association between HAE and elevated ECP levels. Their analyses ultimately supported a stable relationship between HAE and increased ECP.

The importance of this study lies in the fact that it does not merely reiterate the conventional pathophysiological framework of HAE, but rather re-examines disease heterogeneity from the perspective of inflammatory biomarkers. This line of inquiry is highly thought-provoking; however, the generalizability of the findings, the strength of the mechanistic interpretation, and their clinical implications all warrant further consideration.

## 2 The potential value of expanding the inflammatory dimension of HAE

Perhaps the most noteworthy contribution of this study is the suggestion that a low-grade and persistent inflammatory activation may exist even during symptom-free intervals in HAE. Previous investigations of HAE have primarily focused on C1 inhibitor deficiency, dysregulation of the contact system, and excessive bradykinin generation (3). By incorporating eosinophil activation into the discussion, the present study broadens the current understanding of the biological background of HAE. The observation that ECP, rather than eosinophil count, was elevated suggests that the relevant alteration in HAE may reflect cellular activation or degranulation rather than a simple increase in eosinophil number. Conceptually, this is important, as it implies that beyond overt vascular leakage during attacks, HAE may also involve more subtle immune-inflammatory activity during interictal periods.

From a clinical perspective, this observation may also help explain phenotypic variability among patients with HAE. Some patients experience relatively infrequent attacks yet still report a considerable disease burden, whereas others present with comorbid asthma, rhinitis, or food allergy, all of which are conditions associated with eosinophilic inflammation. If elevated ECP indeed reflects a background of subclinical inflammatory activity, it may contribute to a better understanding of the complexity of HAE manifestations and may eventually support more refined risk stratification. As the authors also suggest, these findings may prompt a reconsideration of therapeutic strategies that focus exclusively on the control of acute attacks, while underestimating the potential importance of long-term prevention.

## 3 A cautious interpretation of the conclusions

Although this study raises an appealing hypothesis, the current evidence is better interpreted as indicative of an association rather than proof of mechanism. First, elevated ECP does not establish a causal role for eosinophils in the pathogenesis of HAE. As acknowledged by the authors, ECP may serve as a biochemical indicator of eosinophil-related inflammatory activity, but its diagnostic utility at the individual level remains limited, and the marker is susceptible to pre-analytical variability. In other words, ECP is more appropriately regarded as a clue-generating biomarker than as direct evidence that HAE should be reclassified as an eosinophilic inflammatory disease.

Second, the central pathophysiological mechanism of HAE remains bradykinin-mediated vascular hyperpermeability. The value of this study does not lie in challenging that established paradigm, but rather in suggesting that additional immune-inflammatory signals may coexist on top of it. A more balanced interpretation, therefore, is that eosinophil activation may represent an accompanying, modifying, or secondary inflammatory phenomenon in HAE, rather than a dominant pathogenic pathway that can already be considered established.

## 4 Methodological strengths and limitations

The authors should be commended for their careful methodological design. By applying a causal inference framework, post-stratification, and inverse probability weighting, they sought to reduce selection bias and strengthen the robustness of their estimates in a single-center retrospective real-world setting. Adjustment for age, sex, seasonality, and allergic comorbidities further supports the observed association between HAE and elevated ECP.

However, several limitations remain. The small sample size and single-center design restrict the generalizability of the findings. In addition, HAE with normal C1-INH is clinically and genetically heterogeneous, making it difficult to determine whether similar inflammatory patterns exist across subgroups. Because samples were collected during asymptomatic periods, the study also cannot address dynamic changes in ECP during acute attacks or its relationship with attack severity and disease activity. Moreover, although all samples were obtained during symptom-free intervals, the exact timing of blood sampling in relation to the most recent HAE attack may have varied among patients. Such variation could be important, as different intervals since the last attack may reflect different levels of residual inflammatory activity and may therefore influence ECP measurements. Another limitation is that differences in treatment exposure were not fully addressed. The type of HAE therapy, as well as the duration of treatment, may modify disease activity, attack frequency, and the baseline inflammatory milieu, thereby potentially affecting ECP levels independently of the underlying disease process. Finally, as ECP is sensitive to testing procedures and sample handling, its reproducibility and clinically relevant thresholds still require prospective validation.

## 5 Discussion

Overall, the study by Boch et al. raises an important question: whether hereditary angioedema should be regarded not only as an episodic, bradykinin-mediated vascular disorder, but also as a disease with a background of persistent low-grade immune-inflammatory activity. This perspective is clinically and biologically relevant, as it may encourage further investigation into inflammatory phenotypes, endothelial activation, and comorbidity patterns during symptom-free periods.

However, these findings should be interpreted with caution. Although elevated ECP supports the presence of eosinophil-associated inflammatory signaling, it does not establish a direct role in the core pathogenesis of HAE and is not yet sufficient to inform clinical decision-making. Future studies should focus on multicenter prospective validation, longitudinal comparisons between attack and remission phases, and integrated analyses of ECP with complement-related and endothelial biomarkers, as well as the impact of prophylactic therapies.

Taken together, the main contribution of this study is not to redefine the established pathophysiology of HAE, but to draw attention to a potentially underrecognized inflammatory dimension of the disease. This provides a meaningful basis for future mechanistic and clinical research.
